# Cluster analysis of functional independence in community-dwelling older people

**DOI:** 10.1186/s12877-022-03684-2

**Published:** 2022-12-23

**Authors:** Esther A.L.M. Molenaar, Di-Janne JA Barten, Anne M.S. de Hoop, Nienke Bleijenberg, Niek J de Wit, Cindy Veenhof

**Affiliations:** 1 Research Group Innovation of Human Movement Care, Research Centre for Healthy and Sustainable Living, Utrecht University of Applied Sciences, Utrecht, The Netherlands; 2 Department of Rehabilitation, Physical Therapy Science & Sports, Brain Center Rudolf Magnus, University Medical Center Utrecht, Utrecht University, Utrecht, Netherlands; 3 Research group Innovative Testing in Life Sciences and Chemistry, Research Center for Healthy and Sustainable Living, University of Applied Sciences Utrecht, Utrecht, the Netherlands; 4 Department of General Practice, Julius Center for Health Sciences and Primary Care, University Medical Center Utrecht, Utrecht University, Utrecht, The Netherlands; 5 Center for Physical Therapy Research and Innovation in Primary Care, Julius Health Care Centers, Utrecht, The Netherlands

**Keywords:** Functional independence, Older persons, Subgrouping

## Abstract

**Background:**

The concept of Functional Independence (FI), defined as ‘functioning physically safe and independent from other persons, within one’s context”, plays an important role in maintaining the functional ability to enable well-being in older age. FI is a dynamic and complex concept covering four clinical outcomes: physical capacity, empowerment, coping flexibility, and health literacy. As the level of FI differs widely between older adults, healthcare professionals must gain insight into how to best support older people in maintaining their level of FI in a personalized manner. Insight into subgroups of FI could be a first step in providing personalized support This study aims to identify clinically relevant, distinct subgroups of FI in Dutch community-dwelling older people and subsequently describe them according to individual characteristics.

**Results:**

One hundred fifty-three community-dwelling older persons were included for participation. Cluster analysis identified four distinctive clusters: (1) Performers – Well-informed; this subgroup is physically strong, well-informed and educated, independent, non-falling, with limited reflective coping style. (2) Performers – Achievers: physically strong people with a limited coping style and health literacy level. (3) The reliant- Good Coper representing physically somewhat limited people with sufficient coping styles who receive professional help. (4) The reliant – Receivers: physically limited people with insufficient coping styles who receive professional help. These subgroups showed significant differences in demographic characteristics and clinical FI outcomes.

**Conclusions:**

Community-dwelling older persons can be allocated to four distinct and clinically relevant subgroups based on their level of FI. This subgrouping provides insight into the complex holistic concept of FI by pointing out for each subgroup which FI domain is affected. This way, it helps to better target interventions to prevent the decline of FI in the community-dwelling older population.

## Background

The global aging of the population causes an increase in the prevalence of associated diseases [1]. This increased burden of most chronic diseases results in a vicious circle of limitations in physical performance followed by constraints in (instrumental) daily life activities [2]. To prevent these medical en social consequences and to respond to the accompanying increased pressure on healthcare professionals and the healthcare system, a conceptual change in care for older people is urgently needed. In line with the approach to healthy aging, it is advocated that future care for older people should aim to maintain the functional ability to enable well-being in older age [3, 4].

**Fig. 1:**
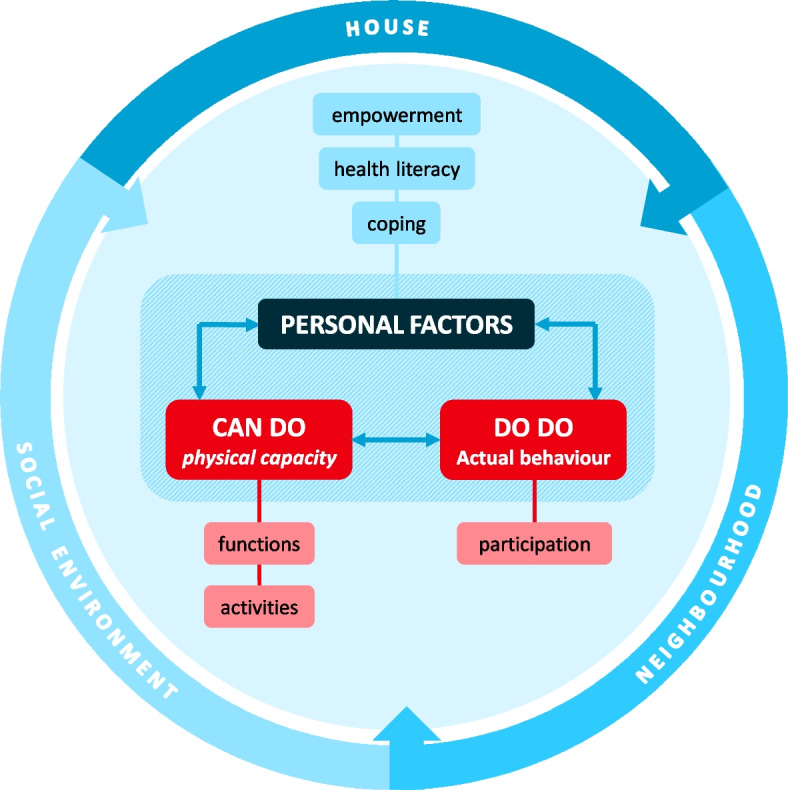
Graphical display of the concept of Functional Independence in community-dwelling older persons. It shows the complexity of compromising the concept of Functional Independence which consists of different constructs

It is advocated that preserving function ability, with proactive instead of reactive care for older people, will lead to the prevention of limitations in daily life activities and maintaining ‘Functional Independence‘ for as long as possible [5]. Functional Independence (FI) is defined as ‘Functioning physically safe and independent from other persons, within one’s context [6]’. The conceptual model of FI, aligned with the International Classification of Functioning, disability, and health (ICF), consists as interpreted by Molenaar et al., of both physical and personal features such as coping, empowerment, and health literacy. In addition safety and contextual factors of the individual contribute to FI. Figure 1 shows the complexity of the individual FI state influenced by the different constructs [6]. This aligns with the definition of health being a dynamic state, in which the emphasis is on the ability to adapt and self-manage, expressed in Fig. 1 by the considerable part of the personal factors (coping, empowerment, and health literacy) [7].

Proactive care in older people and healthy aging include early recognition of older persons at risk of losing their FI [8]. This can be facilitated by monitoring FI among community-dwelling older people and timely referral to an appropriate care professional [9]. However, FI is a dynamic and complex concept in which large individual differences exist. Therefore, it is likely that dividing this population into subgroups will give insight into the domain or domains affected in each group. This may lead to enabling tailored engagement of particular healthcare professionals. In this way identifying clinical subgroups may lead to better treatment and prevention of decline in FI [9, 10].

Therefore, to help healthcare professionals to timely recognize persons at risk of losing FI, and to develop healthcare policy, the identification of subgroups is necessary [10]. In this study, we aimed to identify distinct subgroups of FI in Dutch community-dwelling older adults and describe them according to individual characteristics.

## Methods

### Design and setting

This study has an observational, cross-sectional design [6]. A retrospective analysis of previously collected data for validation of a Core Outcome Set measuring FI (COSFI), in community-dwelling older people in the city of Utrecht, The Netherlands was carried out [11]. Measurements took place in two different districts of Utrecht. Public locations, in the community, that were easily accessible for older persons, such as community centers, fitness centers, and physical therapy practices were used as test locations; persons who were unable to visit the test location were offered a home assessment.

### Participants

Older people were eligible for participation if they met the following inclusion criteria: (a) ≥ 65 years of age and (b) living in the community. As this study focuses specifically on retaining FI and preventing FI decline, we excluded people living in care homes that received institutional care (c). In addition, persons were excluded when they: (d) were unable to understand verbal and written instructions in Dutch or (e) had an insufficient cognitive level to independently complete the required questionnaires. Eligibility of cognitive level was established by the clinical view of the research team; older adults that experienced such severe cognitive problems, that hindered the appropriate completion of the questionnaires, were excluded. Participants were recruited in different ways. District nurses, physical therapists, social workers, and neighborhood sports coaches from the two districts were asked to promote the study among older clients. Additionally, public promotional activities were used to recruit participants for the study (e.g. spreading flyers at supermarkets and community centers, hanging posters in public places, and advertising on local and social media).

### Study outcome

The primary outcome of this study is the validated subgrouping of FI in community-dwelling older people based on COSFI scores. As mentioned previously, FI represents a complex, personalized construct that comprises different domains, including a person’s physical capacity, coping style, health literacy, independence, and fall history [6]. Discriminative validity of the COSFI is moderate for predicting the different levels of FI but good for differentiating FI according to the living situation (i.e. older people in residential care and community-dwelling older people) [11]. The secondary outcome is a description in detail of the derived subgroups of FI combining the COSFI outcomes and demographic characteristics.

The COSFI covers specific domains of FI, also presented in Table 1:

**Table 1:** Content of the specific domains of Functional Independence covered by the Core Outcome Set Functional Independence

Domain of FI	Measurement	Target population
Physical capacity	SPPB	Older adults
	FICSIT-4	Older adults
	JAMAR	General population and community-dwelling older adults
Coping	COFLEX: The COFLEX versatility scale and COFLEX reflective scale	Patients with chronic reumatoïd arthritis
Health literacy	NVS-D	Older adults
Independence	Received professional help; Yes or No	n/a
Fall history	1 or more falls in the past; Yes or No	n/a

*SPPB* Short Physical Performance Battery, *FICSIT*  Frailty and Injuries Cooperative Studies of Intervention Techniques, *JAMAR* JAMAR Hand Held Dynamometer, *TUG* Timed Up and Go test, *COFLEX* COping FLEXibility questionnaire, *NVS-D* Dutch Newest Vital Sign

### Measurements

#### Physical capacity

Physical capacity was operationalized as ‘the ability to perform daily life activities ’ [12]. It was measured by four different measurement instruments, that are complemental in covering the essential components of physical capacity related to the ability to safely perform daily life activities. Furthermore, these instruments are validated in the targeted population of older adults and are feasible in daily practice: The Short Physical Performance Battery (SPPB); this instrument is highly recommended to assess physical capacity in terms of validity, reliability, and responsiveness in community-dwelling older persons [13, 14]. It contains three subscales: balance, gait speed, and lower extremity strength [15]. As a supplement to the SPPB, the Frailty and Injuries Cooperative Studies of Intervention Techniques (FICSIT-4) measurement instrument was added to test static balance more extensively [16]. Additionally, hand-grip strength was assessed by using the hand-grip strength procedure of the JAMAR Hand-Held Dynamometer (JAMAR) [17]. Hand-grip strength is a reliable and valid characteristic to get insight into overall muscle strength in older people [18, 19]. Furthermore, the Timed Up and Go test (TUG) is a valid tool for screening balance deficits in senior citizens [20]. It is an important part of the physical capacity domain of FI since the dynamic balance during walking also plays an important part in functioning independently [21]. The amount of time needed to stand up from a chair, walk 3 meters, walk back to a chair and sit down again is scored, a maximum score of 240 seconds is registered when participants are not capable to execute the TUG test.

#### Coping

Coping concerning FI was interpreted as dynamic cognitive and behavioral efforts to manage a decline in physical function or physical disability [22]. The COping FLEXibility questionnaire (COFLEX) was used to assess coping in the current study. The COFLEX is a tool to measure one’s ability to use both assimilative and accommodative coping strategies to deal with stressors in different situations [23]. The COFLEX is divided into two subscales that were analyzed separately: coping versatility (9 items) and reflective coping (4 items). Coping versatility refers to the ability to switch between using existing knowledge and skills or adapting existing skills and knowledge to deal with a new situation following personal goals and situational demands [23]. For instance: adjustment of physical goals when a chronic disease causes constraints in physical functioning and the existing goal isn’t obtainable anymore [24]. Reflective coping represents someone’s ability to generate different coping options and to make a considered choice in the most suitable coping strategy in a given situation. For example, when the balance of an older person is affected, this person should take this into account in his or her choice of coping strategy in which he or she has to make a considered decision concerning safety in a certain situation.

#### Health literacy

Health literacy is an interaction between the skills of the individual to obtain, process, and understand health information and services necessary to make appropriate health decisions and a requirement of the health system [25]. Health literacy was assessed with the Dutch version of the Newest Vital Sign (NVS-D) [23]. The NVS-D is a valid and reliable screening tool that identifies patients at risk for low health literacy [26]. It contains six questions to assess one’s level of health literacy by determining an individual’s ability to find and interpret information on an ice cream nutrition label.

#### Independence

To monitor the level of independence, a variable concerning receiving help from other persons was added. Because this help can also be convenient and not necessary, we specifically asked if professional help, by which we mean paid help in a person’s home, was received by asking the following question: “ Do you receive professional help?”. This question could be answered by ‘yes’ or ‘no’.

#### Fall history

Safety is an important feature of FI [8]. Functioning safely in the context of daily life includes a minimal risk of falling. As falling in the past year is an important predictor of the risk of falling in the future we added a question concerning falling in the past, which is widely used in fall screening of older people: “ Have you fallen in the past year?” This question could be answered by ‘yes’ or ‘no’ [27].

#### Additional characteristics

Demographic data of the participating older people were collected on age (years), gender (male, female, unknown), and educational level according to the Dutch Central Bureau of Statistics; High (associate degree programs, higher education bachelor programs; Master degree programs, Middle (upper secondary education, (basic) vocational training and middle management and specialist education) and Low (primary and special primary education), multimorbidity (i.e., the presence of more than one chronic disease in an individual) (0–2, > 2 chronic diseases) [28], and living situation. The living situation was subdivided into community-dwelling; living alone, community-dwelling; with someone else, and living in a supported house. This last subgroup refers to a group of older persons whose accommodation is provided alongside support, supervision, or care to help them live as independently as possible in the community. (community-dwelling alone, community-dwelling with a partner, living in a supported house).

### Statistical analyses

Agglomerative hierarchical binary cluster analysis, applying Ward’s method and between-group linkage with squared Euclidean Distance measure, was used to identify clusters of FI in community-dwelling older people [30].

### Data transforming

In preparation for the cluster analysis, the ordinal and continuous outcome scales of the COSFI (JAMAR, SPPB, NSVD, and the COFLEX versatility scale and COFLEX reflective scale) used in the cluster analysis were first converted to binary variables to meet the assumption for similar outcome scales [29]. For a transition from ratio and ordinal variables to binary variables we used the clinical cut-off point; (0 = beneath the cut-off point) (1 = above the cut-off point) [15, 18, 30]. For a transition of the JAMAR outcomes, a subdivision between men and women was made as proposed by Arvandit et al. [31]. When a clinical cut-off point was not available, we used the mean score as a proxy variable; (0 = beneath the mean score 1 = above the mean score).

### Handling missing data

Participants were excluded in case of missing values on one of the clustering variables since the analysis did not allow missing values. Imputation was not performed because the missingness was not severe (< 5%) and the data was Missing Completely at Random (MCAR) [32].

### Assumptions for cluster analysis

Subsequently, assumptions for agglomerative hierarchical binary cluster analysis were investigated. The clustering variables from the COSFI were checked on multicollinearity. If the correlation coefficient (Pearson r) was ≥ 0.90, the variable was excluded from the cluster analysis [33]. Furthermore, the assumption for similar outcome scales was met as we converted the different outcome scales to binary variables.

### Model fitting

To identify distinct clusters, first, a visual assessment of the dendrogram and the agglomeration schedule was performed by two researchers (EM, JB). The visual representations of the cluster analysis, the hierarchical dendrogram, and the agglomeration schedule were inspected individually. The results from the dendrogram and the agglomerative schedule, showed the change in the agglomerative coefficient, indicating possible cluster solutions in which clusters indicated the best similarities and deviations on domains of FI. The graphical display shows the distance within the clustering algorithm until joining the next cluster. Possible plausible numbers of cluster solutions were selected by the two first authors.

### Validation of the cluster solution

To determine to what extent the derived clusters are distinguishing, we examined scores on domains of FI per derived cluster. This analysis comprised a between-cluster comparison of COSFI scores on each domain to examine if the number of clusters chosen by the researchers has resulted in clinically distinctive groups concerning the results of the measurements included in the COSFI. Chi-square tests were used to examine differences in scores on domains of FI between the different clusters.

### Sensitivity analyses

This analysis comprised a between-cluster comparison of participant demographics to examine differences in population characteristics between subgroups of FI. One-way ANOVA was used to compare the mean in age and BMI, between different subgroups of FI. If data were not normally distributed, an alternate parametric test was obliged. The categorical outcomes (educational level, and living situation) were compared by using the Kruskal Wallis test and Chi-square tests were used for nominal data (gender and co-morbidity).

An overview of participant demographics and outcomes of the cluster analysis was created to display the different subgroups in FI.

All statistical analyses were performed in IBM SPSS Statistics (version 25, IBM Corp., Armonk, N.Y., USA).

### Sample size

Because this study was a retrospective analysis of existing data within an overarching research project, we used a convenience sample of all participants available meeting the inclusion criteria (*N* = 153). Clear guidelines regarding the minimum sample size in cluster analysis are lacking [32]. The goal of this cluster analysis is to construct clinically relevant clusters, so the number of variables should be proportional to the sample size. We used a commonly accepted rule of thumb to estimate the desired number of participants [34]. The use of eight different cluster variables led to an estimated number of 2^8^= 256 Participants. When a variable is excluded due to a correlation coefficient of ≥ 0.90 an estimated number of 2^7^ leads to an estimated number of 128 [35].

### Ethics

Ethical approval for the study was obtained by the Research Ethics Committee of Utrecht University of Applied Sciences (reference number 85_000_2019). No personally identifying data were incorporated into the dataset. Informed consent forms were signed by participants before study participation. The whole test procedure took 60 min, participants were guided through the test procedure and were offered help filling in the questionnaires. Participants were allowed to rest or take a break whenever they wanted.

## Results

### Participant demographics

A total of 153 community-dwelling older persons were included for participation in the study. Participants’ age ranged from 65 to 95 years (79 ± 7.5 years) with 67% identified as female. Demographic characteristics and outcomes of COSFI measures are shown in Table 2.

**Table 2:** Characteristics of participants

Characteristics	(*n* = 153)
Age *(yr.)*, mean (SD)	79 (7.5)
Gender (%)	
Male	41
Female	59
Unknown	0
Receiving professional help, yes (%)	54
Educational level (%)	
High	17
Middle	35
Low	48
Number of Falls, mean (SD)	1.5 (0.7)
BMI (kg/m2); mean (SD)	28.9 (7.1)
Co-morbidities (% >2)	73
Living situation (%)	
Community-dwelling alone	63
Community-dwelling with a partner	37
JAMAR, mean (SD)	29.6 (11.7)
Male	38.9 (11.3)
Female	23.3 (6.7)
NVSD, median (IQR)	3 (3)
TUG, median (IQR)	9.9 (8.9)
COFLEX V, median (IQR)	27 (9)
COFLEX R, median (IQR)	11.5 (4)
SPPB, median (IQR)	9 (4)
FICSIT-4, mean (SD)	18.4 (7.3)

*SD *Standard Deviation, *IQR* InterQuartile Range, *JAMAR* JAMAR hand held dynamometer, *NVS-D* Dutch Newest Vital Sign, *TUG* Timed Up and Go test, *SPPB*  Short Physical Performance Battery, *COFLEX* COping FLEXibility questionnaire; COFLEX V versatility scale and COFLEX R reflective scale, *FICSIT* The Frailty and Injuries Cooperative Studies of Intervention Techniques

### Identified subgroups of FI in community-dwelling older people

The pre-analysis check for multicollinearity showed a high correlation between the outcomes of the FICSIT-4 instrument and the SPPB (r ≥ 0.90). We chose to include the SPPB since the SPPB is more extensive and covers multiple domains in contrast to the FICSIT-4 which only covers the balance domain. Therefore we excluded the FICSIT 4 outcome from the hierarchical cluster analysis.

**Fig. 2:**
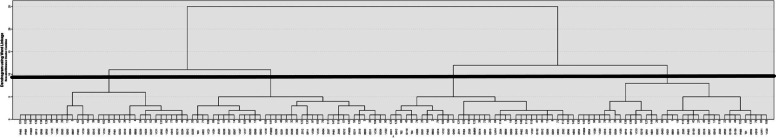
Dendrogram hierarchical cluster analysis of the Core Outcome Set of Functional Independence in community-dwelling older persons. A horizontal line pierces the four clusters of the most appropriate cluster solution

The visual inspection of the dendrogram of the hierarchical cluster analysis showed from top-down two large clusters. The research team considered two clusters not to be clinically feasible since the dichotomy would not be specific enough to guide clinical follow-up. Therefore consensus was reached on a 4-cluster solution derived from the first two clusters being the most appropriate cluster solution [35] (Fig. 2); a horizontal line through the dendrogram on the number of options shows the most clinically plausible solution considering diversity between persons in aspects of FI.

**Fig. 3:**
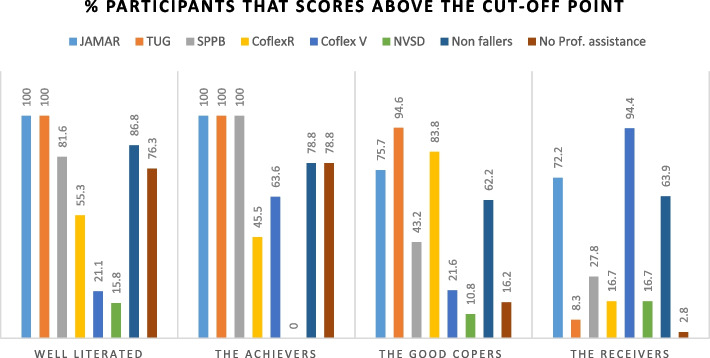
Graphical display of achieved scores on cluster variable per subgroup. It shows the percentage of participants in the cluster that scores above the cut-off point on the cluster variables from the Core Outcome Set of Functional Independence

Clusters are rated from highest to lowest FI level and labeled according to characteristics (Fig. 3 and Table 3).

**Table 3:** Description and names of the different subgroups as a result of the hierarchical cluster analysis on variables concerning Functional Independence

Cluster	Characteristics	Name
Cluster 1	Physically strong, Well Informed and educated, independent, non-faller, limited reflective coping style	The Performers - Well Informed
Cluster 2	Physically strong however a limited versatility coping style and health literacy level.	The Performers - Achievers
Cluster 3	Physically somewhat limited, sufficient coping styles, however, do receive professional help	The Reliant- Good Copers
Cluster 4	Physically limited, insufficient coping styles, receives professional help. Least level of functional independence in total.	The Reliant - Receivers

### Subgroups 1 and 2: the Performers

Persons in cluster 1 score overall the best on all aspects of FI, they show scores above the mean or cut-off point on all measurements except for the COFLEX versatility scale. The average older person in cluster 1 does not fall or receive professional help in addition, there are only male older persons in this subgroup. Both clusters 1 and 2 score well on the physical outcome measures, and therefore are mentioned as The Performers, however, this group has an average sufficient health literacy level and therefore is named the Well informed.

The average scores on the physical measurements in cluster 2 are above the cut-off points. Furthermore, most persons in cluster 2 like in subgroup 1 do not fall nor receive help. Also, there are more male older people than females in this group. However, they score below the mean score of the COFLEX reflective scale and the cutoff point of the NVSD measurement tool for health literacy. Because of the overall adequate achievements on the physical performance measurement tools, this group is called the Achievers.

### Subgroups 3 and 4: the Reliant

In cluster 3, the SPPB is scored mostly below the cut-off point. Furthermore, more than 97% of the persons in this cluster receive professional help, and therefore this group is mentioned together with subgroup 4 as The Reliant. On the other hand, the average person in cluster 3 scores above the mean score on the COFLEX reflective scale. Hence, the name of the subgroup is The Good Copers.

Cluster 4 is characterized by the large number of persons receiving professional help. Furthermore, scores are mostly below the average and cut-off points which makes it the cluster with the lowest level of FI of the four clusters. As they are most likely to receive professional help at the moment and in the future, this subgroup is named The Receivers.

### Validation of the cluster solution

To determine to what extent the derived clusters are distinct, we examined significant differences in COSFI outcomes between subgroups of FI. This analysis showed a significant difference between the established clusters in COSFI measurement scores. Table 4 shows significant (*p* ≤ 0.05) differences for 7 out of 8 measurement outcomes of the COSFI. Only the Timed Up and Go test did not differ significantly between the four groups.

**Table 4:** Differences between groups in measurement outcomes of the Core Outcome Set Functional Independence

Domain of FI	Measurement	Cut-off points	Subgroups of Functional Independence			
			**Well informed**	**The achievers**	**The good copers**	**The receivers**
			*N* = 38	*N* = 33	*N* = 37	*N* = 36
Physical capacity	JAMAR^*^	Above	38	33	28	26
		Below	0	0	9	10
	SPPB^a*^	Above	31	33	16	10
		Below	7	0	21	26
	TUG	Above	38	33	35	33
		Below	0	0	2	3
Coping	COFLEX V^*^	Above	17	18	31	6
		Below	21	15	6	30
	COFLEX R^*^	Above	8	21	8	34
		Below	30	12	29	2
Health literacy	NVSD^*^	Above	32	0	4	6
		Below	6	33	33	30
Independence	Receiving professional help^*^	No	29	33	6	1
		Yes	9	0	31	35
Fall history	Falling^*^	No	33	26	23	23
		Yes	5	7	14	13

*NVS-D* Dutch Newest Vital Sign, *TUG*  Timed Up and Go test, *COFLEX* Coping Flexibility questionnaire, *V*  Versatility scale and *R* Reflective scale, *SPPB* Short Physical Performance Battery.

^*^significant difference between groups *p* = < 0.05

### Sensitivity analyses

Significant differences (*p* ≤ 0.05), in participant demographics, between all groups were found in age and co-morbidities. In addition, a significant difference (*p* ≤ 0.05) was found in the education level between clusters 1 and 2, and 1 and 4 (Table 5).

**Table 5:** Differences in participant demographics between the four clusters of Functional Independence

Demographics	Clusters			
	**Well Informed**	**The Achievers**	**The Good Copers**	**The Receivers**
	*n* = 38	*n* = 33	*n* = 37	*n* = 36
Age *(yr)*, mean (SD)	75.6 (6.7)*	76.7 (6.6)*	81.8 (6.3)*	82.8 (7.3)*
Gender (% Male)	100	74	54	50
BMI (kg/m2), mean (SD)	29.1(6.3)	29.8(7.0)	26.7(6.2)	30.5(8.4)
Educational level (%)				
High	42**	11**	8	8**
Middle	32**	24**	49	36**
Low	26**	64**	43	56**
Co-morbidities, yes (%)	55*	67*	76*	89*
Living situation (%)				
Community-dwelling alone	50	55	65	50
Community-dwelling with a partner	48	45	30	31
Community-dwelling living in a supported house	2	0	5	19

*significant difference between groups *p* ≤ 0.05, **educational level differs significantly (*p* = 0.05) between clusters 1 and 2, and 1 and 4

## Discussion

The primary aim of the study was to identify clinically relevant subgroups of functional independence in community-dwelling older adults. As FI consists of different domains in which different professionals are requisite, the division into subgroups could provide insight into who might need help in which domain. Hierarchical cluster analysis identified four distinctive subgroups of FI in community-dwelling older adults. These subgroups showed significant differences in clinical COSFI outcomes and were distinct from each other regarding demographic characteristics like age, gender, educational level, and presence of co-morbidity, which makes the subdivision also clinically relevant.

### Comparison with literature

The results of our study and the established subgroups are in line with recent literature determining the relation between age, falling, co-morbidities, and increased demand for help [36, 37]. In addition, other studies show similarities between the reliant groups and frail older people. In particular, the group that is most dependent on assistance shows similarities in terms of physical outcomes as well as coping and health literacy outcomes in comparison with frail older people [38]. It underlines the important role of physical status in the concept of FI [11]. In addition, the absence of female persons in the Well-informed group is remarkable. However, as female older persons show a larger decrease in functional status decline, it is likely that this group which scores the best on the functional performance tests, is dominated by males [39]. Moreover, earlier studies demonstrated the relationship between educational level and health literacy level, as older female persons are overall less educated this could also explain the diminished presence in addition low health literacy is associated with poorer health outcomes [40].

### Clinical implications

Furthermore, this study shows the complex interaction between physical and cognitive or behavioral factors in maintaining FI. For instance, results show that persons scoring better on health literacy outcomes, but lower on the SPPB (the Well-informed group) are less likely to fall than persons in the Achievers’ group who score better on all physical outcomes. Moreover, the Performers-achievers score so well on the physical performance tests that they may not need help with physical functioning. However, this group might benefit from help in coping when limitations are experienced. A different kind of health care professional will therefore have to be involved with persons in this group instead of persons as in the reliant-Good Coper group in which physical limitations are the most important issue. These differences between subgroups in performance on clinical outcomes underline the importance of the holistic view of the FI concept and the relevance of establishing subgroups in FI based on the COSFI outcomes. Furthermore, it encourages inter-professional collaboration around this topic [6].

### Strengths & limitations

Although a larger sample size is preferable, we made a well-considered choice of using hierarchical cluster analysis with Ward’s method as this analysis is most suitable and frequently used in a small sample size [33, 34, 41]. In addition, we converted variables to a similar binary scale to prevent one of the outcome measures from being a dominant factor in the analysis. To convert these variables we utilized clinical cut-off values [42]. By choosing a clinical cut-off point we argue that the outcomes became clinically interpretable which makes it a study strength. However the COFLEX did not have a clinical cut-off point, we chose the pragmatic solution by dividing persons scoring above and below the mean value on both the subscales of this measurement instrument to create a binary scale. It could be argued that this was the right solution. The mean value does indicate the result for the overall group. Though, by using the mean value we created a fictive cut-off value that does not indicate persons scoring below the mean have inadequate coping behavior. These outcomes should be interpreted with caution, therefore.

Furthermore, the process of choosing the most appropriate number of clusters based on the dendrogram is a subjective matter. Authors could have had a preference for a practical appropriate number of clusters to be feasible in healthcare practice. However, they analyzed the dendrogram individually and came to a similar choice.

We found significant differences in all COSFI outcome measurements in the cluster analysis; JAMAR, SPPB, COFLEX V, COFLEX R, and the NVSD, between the different subgroups. This strengthens the choice for the four-cluster solution. The only exception was outcomes on the TUG. However, the choice of the cut-off point for these measurements could be debated. The participants in our study generally did not reach this cut-off point (only 5 out of 121 did ) which may have caused bias in the interpretation of this outcome measure when treated as a binary measurement [43, 44]. However it should be part of the COSFI considering the COSFI could also be used for measuring older persons with fewer FI levels, for instance, not community-dwelling.

Finally, as older persons with cognitive impairment were not able to complete the COSFI we chose to exclude these persons from participation in the study. However, we are aware of the influence of cognitive impairments on a person’s level of independence and thus on the level of FI. As physical status and performance are centralized in FI, cognitive impairment is disregarded in this specific study.

### Recommendations

FI is a complex concept, resulting from the interaction between physical cognitive, and behavioral elements, with different outcome measures [6]. To estimate prognosis and to personalize interventions, it is helpful and practical to allocate persons in subgroups according to differences in outcome measures. In this way, the targets for interventions are better defined and consequently, it helps to decide which healthcare professional should be engaged [45]. Further research investigates the practical relevance and recognizability of the four clusters and which cluster matches which caregiver [46].

The next step is to develop appropriate recommendations for the follow-up of each subgroup, in collaboration with multidisciplinary professionals and the target population. These recommendations could be used as guidance for decisions in the cure and care of individual persons to provide patient-centered care. In addition, these should be added to the needs of the individual and tailored by the attending care professional [47].

Furthermore, longitudinal studies are indicated to; track the change of functional independence over time, validate the cluster results, and monitor the development of the different variables. This could give us insight into the most important indicator(s) for losing FI, which would allow us to intervene rapidly when the risk of losing FI occurs.

## Conclusion

Community-dwelling older persons can be allocated to four distinct and clinically relevant subgroups based on their level of Functional Independence. This subgrouping provides insight into the complex holistic concept of Function Independence and helps to better target interventions to prevent the decline of functional independence in the community-dwelling older population.

## Data Availability

The dataset analyzed during the current study is not publicly available due to further data collection during the ongoing overarching research project but is available from the corresponding author upon reasonable request.

## Abbreviations

FI: Functional Independence
COSFI: Core Outcome Set Functional Independence
SPPB: The Short Physical Performance Battery
FICSIT-4: The Frailty and Injuries Cooperative Studies of Intervention Techniques
TUG: The Timed Up and Go test
COFLEX: The COping FLEXibility questionnaire
NVS-D: The Dutch version of the Newest Vital Sign
MCAR: Missing Completely at Random
ICF: International Classification of Functioning, Disability and Health
